# Intermediate hosts of *Protostrongylus pulmonalis* (Frölich, 1802) *and P. oryctolagi* Baboš, 1955 under natural conditions in France

**DOI:** 10.1186/s13071-015-0717-5

**Published:** 2015-02-15

**Authors:** Célia Lesage, Cécile Patrelle, Sylvain Vrignaud, Anouk Decors, Hubert Ferté, Damien Jouet

**Affiliations:** EA 4688 (VECPAR), UFR de Pharmacie, Université de Reims Champagne-Ardenne, 51 rue Cognacq-Jay, 51096 Reims, France; Office National de la Chasse et de la Faune Sauvage, Direction des études et de la recherche, 5 rue de Saint Thibaud, 78610 Auffargis, France; USR 3278 CNRS-EPHE-Université Perpignan, Perpignan, France

**Keywords:** Protostrongyliasis, Lagomorphs, Snails, Intermediate hosts, Molecular identification, Epidemiology

## Abstract

**Background:**

*Protostrongylus oryctolagi* and *P. pulmonalis* are causative agents of pulmonary protostrongyliasis in Lagomorphs in France. These nematodes need usually one intermediate host for its life cycle, a terrestrial snail. However, some studies, mainly in experimental conditions, have identified the species of snails acting as intermediate hosts.

**Methods:**

In total, 3315 terrestrial snails and 307 slugs were collected in the field in South-Eastern France and analyzed to detect the presence of parasites. Identification of nematode parasites and snails were performed according to morphological and molecular approaches (D2 domain of the 28S rDNA for parasites; 18S and ITS-1 rDNA, COI and 16S mtDNA for snails).

**Results:**

Eighteen snails were found positive for Protostrongylids larvae. Haplotypes of the larvae corresponding to sequences of *P. oryctolagi* and *P. pulmonalis* were detected. Morphological identification of molluscs based on shell characters revealed 4 different morphotypes, and molecular results confirm the membership of these gastropods to the Hygromiidae and revealed 4 different species: *Candidula gigaxii,* 2 species of *Cernuella* sp. and *Xeropicta derbentina*. All infested snails were collected in wine cultures.

**Conclusion:**

This study displays the first description of intermediate hosts of *P. oryctolagi* and the first report of *X. derbentina* as natural intermediate host of *P. pulmonalis.*

## Background

The superfamily of Metastrongyloidea is composed of about 181 species divided into seven families, most of which uses gastropods (generally terrestrial) as intermediate hosts in their life-cycle for the infestation of definitive hosts [1,2].

Since the discovery of the role of snails in the development of *Muellerius capillaris* by Hobmaier and Hobmaier in 1929 [3], several studies have been implemented in order to identify intermediate hosts and to describe the infestation pathways. Life cycle of Angiostrongylidae has been largely studied, because of its involvement in a human infestation [4-6]. The intermediate hosts of Protostrongylidae of domestic ruminants, parasites involved in diseases affecting pulmonary tract of livestock and responsible for economic losses [7], have also been well studied [8-12]. In contrast, developments of Protostrongylidae of Lagomorphs have been studied only in USA [13,14]. Yet, pulmonary protostrongyliasis, disease caused by these nematodes, is frequently encountered and occasionally implicated in the cyclic decline of hare populations in Europe [15,16].

Life-cycle of Protostrongylidae includes a pulmonary adult stage in the definitive host. After emission, the first-stage larvae rejoins the intermediate host, snail or slug, by active or passive penetration [8,9,17-24]. Unlike the trematodes, first-stage larvae of Protostrongylidae migrate to the muscle of foot where they grow to the third-stage larvae in 25 to 30 days, depending on the external temperature [25,26]. Several land snails and slugs have previously been identified as intermediate hosts of Protostrongylidae [1,27,28]. Using experimental approaches, the authors considered that very few species of snail and slugs are refractory to infection by Protostrongylidae. However there are susceptibility differences depending on species, age, density, activity period of gastropods and mobility of first-stage larvae [29-33]. Actually, young of large size snail species with a life of several years were more susceptible to infection than adults and reverse for little species with a life of one year [34,35]. In addition, a same snails’ species can be implicated both in the life cycle of *Protostrongylus* parasiting hares, and in the cycle of other parasites. For example, the snail species *Oxyloma elegans* (Risso, 1826) can be infested by *P. pulmonalis* (parasite of Lagomorphs) and *Muellerius capillaris* (parasite of small ruminants) [28].

All larvae stages of species of the same genus and even of different genera may be morphologically indistinguishable. Indeed species identification using morphological features is only possible on male adult worms [1]. Hence, to obtain a diagnostic tool to differentiate first-stage or third-stage larvae of Protostrongylidae, a molecular approach must be privileged. Among available molecular markers, several studies on nematode species investigating specific identifications used ribosomal DNA, essentially the internal transcribed spacers (ITS1 and 2), known for their inter-specific variability [36-40], and also D2 domain (part of 28S) [41].

Similarly, species identification of snails is not so trivial, when based essentially on morphological criteria. The morphological polymorphism, inter and intra-specific, is extremely important in terrestrial snails depending on many parameters such as age, season, environmental conditions and biochemical factors [42-47]. Recently, using of molecular tools, by studying domains such as internal transcribed spacer and 18S rDNA or the COI and 16S mDNA, have facilitated the recognition of snails at a specific level. The combination of the morphological and molecular approaches has helped with the identification of snails difficult to distinguish purely on morphological criteria or only characterizable by specialized malacologists [48-55].

In France, a recent study conducted on hares identified two species of pulmonary parasite adults: *P. (protostrongylus*) *oryctolagi* Baboš, 1955 and *P.* (*pulmostrongylus*) *pulmonalis* (Frölich, 1802) [41]. Concerning their life cycle, several intermediate hosts of *P. pulmonalis* were previously reported: *Oxyloma elegans* (Risso, 1826), *Pupilla muscorum* (Linnaeus, 1778), *Vallonia tenuilabris* (Braun, 1843) and *Vertigo alpestris* Alder, 1838 [1,28]. However, intermediate hosts of *P. oryctolagi* have never been studied neither in natural nor in experimental conditions.

The aim of our study is to investigate and identify the intermediate hosts of Protostrongylids in natural conditions, by a morphological and molecular approach of parasites and snails, to determine the intensity of infestation and the conditions necessary for the development of these parasites in order to better understand their life cycle and assess risk of contamination for hares’ populations.

## Methods

### Collection

**Figure 1:**
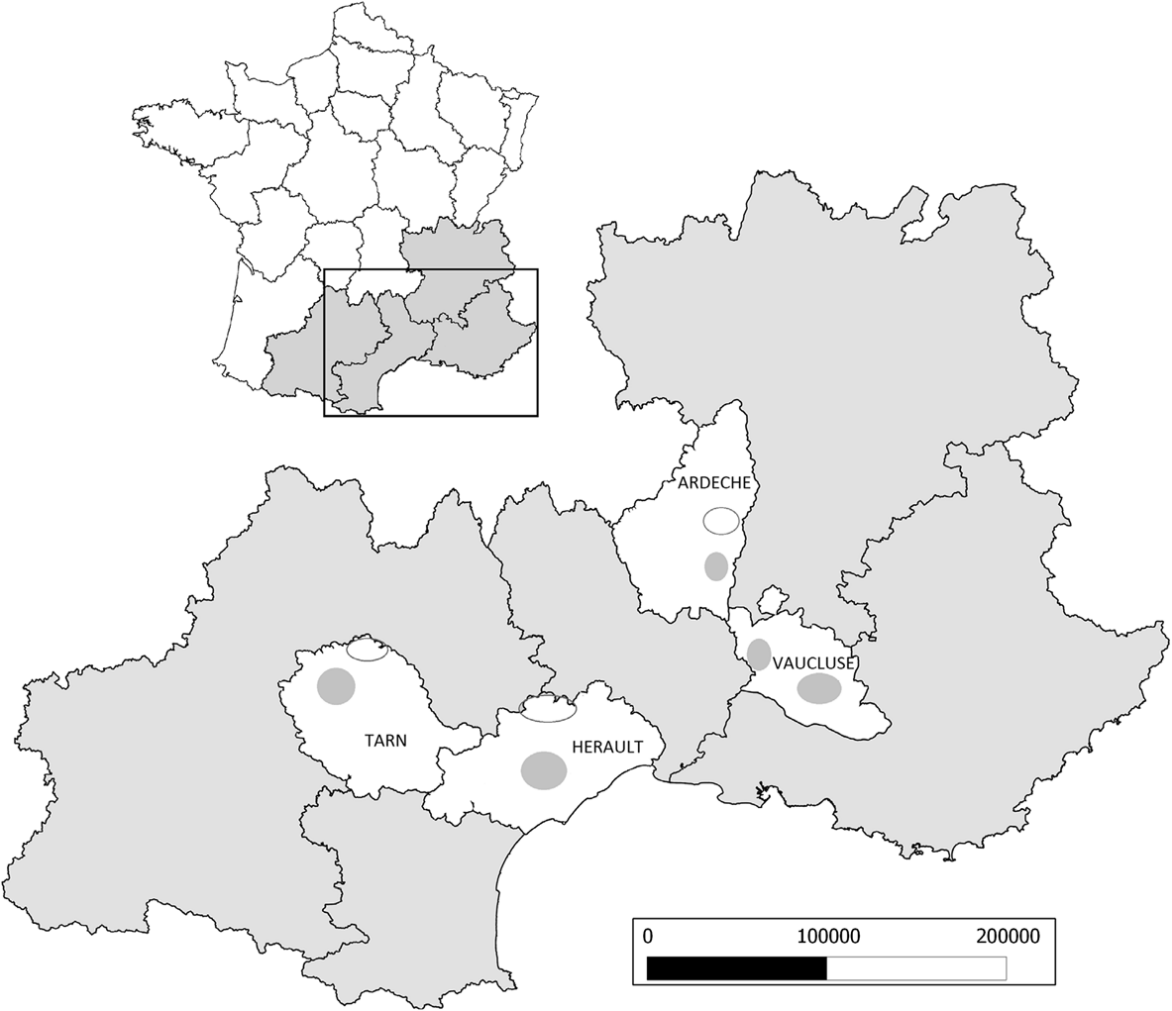
**Map of study sites divided into four departments (Ardèche, Hérault, Vaucluse and Tarn) and composed two well represented habitats: open grassland (empty circle) or wine culture (gray full circle).**

Living gastropods (snails and slugs) were collected from eight areas of 100 hectares in the South-eastern France (Figure 1). The sampled sites were selected according to high density of gastropods and hares according to results previously obtained [41], and located in two open environments known to be favorite habitats of hares: grazed grasslands and wine cultures. On each area, samples were realized on 15 circular points of 1 m^2^. For wine areas, points were located in center or at edge of culture. On each point, a raised wooden plank was disposed on one side, providing shelter for snails when it is warm and causes their gathering under it [56]. Collections were implemented monthly from September to November in 2012 and from March to November in 2013 (except in summer, when snails are inactive and buried). The day of the visit was chosen to be a rainy one or after a rainy one, between 7.00 a.m and 9.00 a.m.

### Parasite examination

Gastropods collected under planks or on plot, were examined for lungworm larvae. All were stored in separate labeled plastic boxes with ventilation openings and refrigerated (4°C) for a maximum of three weeks. Except for the operculated snails the gastropods were drowned in an airtight jar full of water to kill them and involved the output of the foot out of the snail shell. Foots were recovered and crushed between two heavy glass slides to spread the snail tissue for observation with a stereo-microscope [34,57].

Larvae were observed in some snails in the musculature of the foot. After pre-identification on simple morphological criteria (size, sheath, darkened cuticle) as described by Boev (1984) [1], some larvae were extracted out of muscle, which was easy after dissection or pressing between slide and cover slip. Then they were preserved in 90° ethanol for molecular analysis (Table 1).

**Table 1:** **Isolates of larvae and snails from this study used for molecular analysis**

**Snails**								**Parasite**	
**Ref.**	**Origin**	**Morphological identif.**	**Molecular identif.**	**18S**	**ITS-1**	**COI**	**16S**	**Species**	**D2**
EV1	Vaucluse	Morphotype A “white snails”	*Xeropicta derbentina*	KP335095	KP335101	KP335107	KP335113	*Protostrongylus oryctolagi*	KP335119
EV2	Vaucluse		*Xeropicta derbentina*	KP335096	KP335102	KP335108	KP335114	*Protostrongylus pulmonalis*	KP335120
ET3	Tarn	Morphotype B “Cernuella”	*Cernuella* sp.	KP335097	KP335103	KP335109	KP335115	*Protostrongylus oryctolagi*	KP335121
AT10	Tarn		*Cernuella* sp.	KP335098	KP335104	KP335110	KP335116	*Protostrongylus oryctolagi*	KP335122
EA1	Ardèche	Morphotype C “Candidula”	*Candidula gigaxii*	KP335099	KP335105	KP335111	KP335117	*Protostrongylus oryctolagi*	KP335123
ET1	Tarn		*Candidula gigaxii*	KP335100	KP335106	KP335112	KP335118	*Protostrongylus oryctolagi*	KP335124
AA550	Ardèche	Morphotype D “Helicella”	nc	nc	nc	nc	nc	*Protostrongylus oryctolagi*	nc

After rinsing larvae in distilled water, then drying in an oven at 37°C, DNA extraction was performed with a Qiamp DNA mini kit (Qiagen, Hilden, Germany) according to the manufacturer’s instructions. Sequencing of the D2 domain of the 28S was used for the identification of nematodes as described previously [41,58] (Table 2). PCR products were directly sequenced in both directions with the primers used for DNA amplification (Genoscreen, France). Sequence alignment was performed using the ClustalW routine included in the MEGA version 5 software and checked by eye.

**Table 2:** **Primers and conditions used for molecular analyses of larvae and snails (according to Lesage et al., 2014 and Steinke et al., 2004** [41,60]**)**

**Primer**	**Sequence (5′-3′)**	**Initial denaturation**	**40 cycles (denaturation/annealing/extension)**	**Final elongation**
D2 universal	GAAAAGAACTTTGRARAGAGA	1 cycle of 3 min at 94°C	94°C for 30 s/40°C for 1 min/68°C for 1 min	1 cycle of 10 min at 68°C
(Protostrongylids)	TCCGTGTTTCAAGACGGG			
COI universal	GGTCAACAATCATAAAGATATTGG		94°C for 30 s/48°C for 1 min/68°C for 1 min	
(Mollusc)	TAAACTTCAGGGTGACCAAAAAATCA			
16S universal	CGGCCGCCTGTTTATCAAAAACAT		94°C for 30 s/48°C for 40 s/68°C for 40 s	
(Mollusc)	GGAGCTCCGGTTTGAACTCAGATC			
18S specific	CTGGTTGAT(CT)CTGCCAGT		94°C for 30 s/52°C for 30 s/68°C for 40 s	
(Mollusc)	CTGAGATCCAACTAGGAGCTT			
ITS-1 specific	TAACAAGGTTTCCGTAGGTGAA		94°C for 30 s/52°C for 30 s/68°C for 1 min	
(Mollusc)	GCTGCGTTCTTCATCGATGC			

Haplotypes of the larvae were compared to sequences of Protostrongylinae available in GenBank: *P. pulmonalis* (EU595590), *P. pulmonalis* (KJ450993-1018), *P. oryctolagi* (KJ450993-1018), *P. boughtoni* (EU595595), *P. rushi* (EU595598), *P. stilesi* (EU595599), *P. rufescens* (EU595600) and *P. rupicaprae* (EU595601).

### Gastropods identification

Shells of positive and negative snails were preserved and primarily identified on morphological and morphometric features according to the collection of continental gastropods, available at the National Museum of Natural History of Paris [59]. In addition, for positive gastropods infested by nematode larvae, a part of foot was conserved in 90° ethanol for molecular analysis (Table 1). DNA extraction of snail was performed using the same method as for nematode larvae. Sequencing of the ITS-1 and 18S of rDNA and mitochondrial COI and 16S were performed using primers and conditions described by Steinke *et al.* (2004) (Table 2) [60]. Polymerase chain reaction (PCR) was performed in a 50 μl volume using 5 μl of DNA, and 50 pmol of each of the primers. The PCR mix contained (final concentrations) 10 mM Tris HCl (pH 8.3), 1.5 mM MgCl_2_, 50 mM KCl, 0.01% Triton X 100, 200 μM dNTP each base, and 1.25 units of *Taq* polymerase (Eppendorf, Germany). Our sequences were compared with sequences of terrestrial snails available in GenBank for all these domains. Our sequences were deposited in GenBank under accession numbers KP335095 to KP335124.

Phylogenetic trees were constructed using the Neighbor Joining (NJ), the Maximum Likelihood (ML), and Minimum Evolution (ME) methods using the MEGA 5 software [61]. For all NJ, ML, and ME analyses, the most appropriate nucleotide substitution model was determined, gaps were treated as missing data and internal node support was assessed by bootstrapping over 500 replicates.

## Results

### Prevalence

In total, 3622 gastropods were analysed, including 3315 terrestrial snails and 307 slugs. Gastropods were separated depending on their origin, grassland or wine crops. Morphological approach doesn’t allow identifying snails at specific level but only at the family or sometimes at the generic level. They belong to two groups and eight families: Cyclophoridae (Prosobranchia) and, from Pulmonata, families of Chondinidae, Clausiliidae, Enidae, Helicidae, Hygromiidae, Vitrinidae and Zonitidae. Out of the 3315 snails, no Prosobranchia were positive for parasites. Eighteen Pulmonata were positive (0.54%), all originated from wine cultures. A total of 123 third-stage larvae were observed and the number of larvae per snail varied from 1 to 52 with an average of 7 larvae.

Identification of slugs was evoked at family level [62,63]. Identification of slugs brings out four families: Agriolimacidae, Arionidae, Limacidae and Milacidae. None slug was positive for protostrongylids larvae.

### Identification of larvae

**Figure 2:**
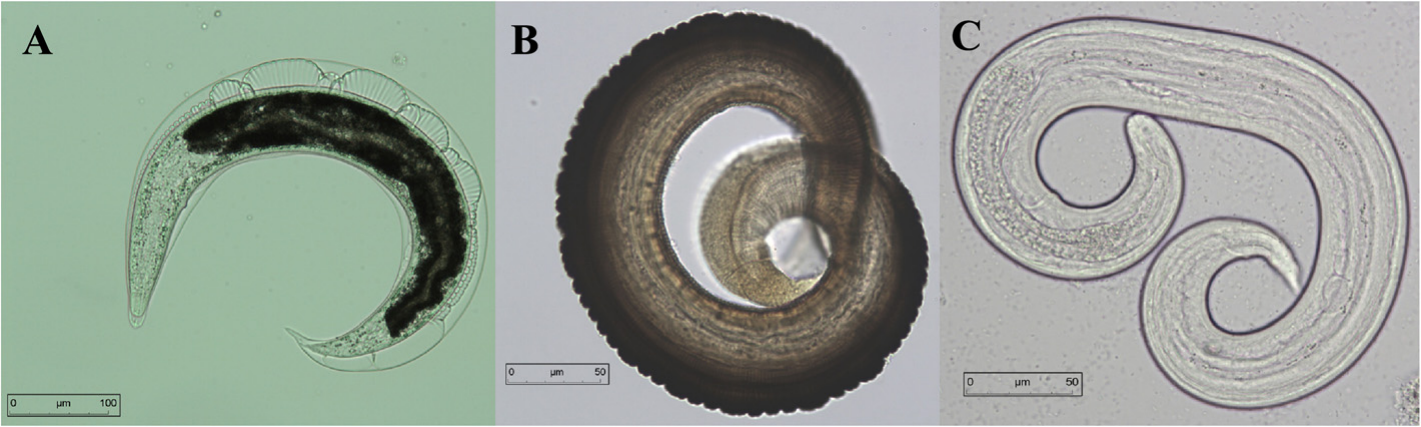
**Different stages of infective larvae of** ***Protostrongylus oryctolagi*** **extracted from snail’s muscle, with external and internal sheath (A), without external sheath (B) and without darkened cuticle (C).**

Pre-identification of larvae of Protostrongylids was based on simple morphological criteria. The third stage (infective) larva of this group is generally covered by a double sheath. The upper sheath is thin, transparent, and the lower sheath is compact and rugose with rough traverse folds of brownish color. The larvae along with its sheath usually coil into a ring. (Figure 2) [1,12].

Larvae were then analyzed using molecular markers (D2) in order to identify them at the specific level. For seventeen positive snails, sequences of the larvae are 100% homologous with the haplotype of *P. oryctolagi*, whereas one snail was positive for larvae whose haplotype corresponds with *P. pulmonalis* deposited in GenBank.

### Identification of positive snails

By pre-morphological identification based solely on shell, the 18 positive snails were all included in the Helicidae and Hygromiidae families, and separated into 4 groups corresponding to their morphotype: A) the first group of white snails including genera such as *Xeropicta* and *Theba* (8 snails); B) a group including snails with morphological features of the genus *Cernuella* (7 snails); C) a group including snails which morphotype corresponds with the genus *Candidula* (2 snails); D) the last group with one postive snail whose morphological criteria seems to correspond to the *Helicella* genus. Specimens of each group were found infected with *P. oryctolagi* larvae, except snails in group A in which the two species of parasites were found.

For each group, two snails were used for molecular analyses (A: sequences EV1 and EV2; B: seq. AT10 and ET3; C: seq. EA1 and ET1), except for the *Helicella* group where tests failed. We did not take into account this snail for our next results.

**Figure 3:**
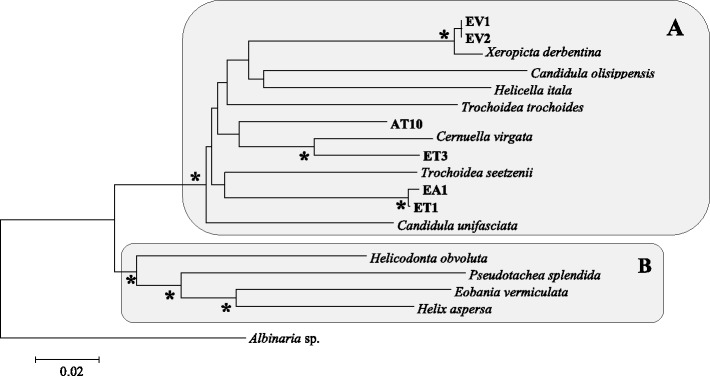
**Phylogenetic tree based on a combined dataset of COI, 16S, 18S and ITS-1 sequences with a total of 1677 nucleotide sites constructed using the Maximum Likelihood method and the general time reversible model (GTR + I + Γ).** The tree has been rooted using *Albinaria* sp. (AY546262/AY546342/AY546382/AY546302). The asterisk indicates bootstrap values of >95 for NJ, ML and ME. **A** = Hygromiidae; **B** = Helicidae.

**Figure 4:**
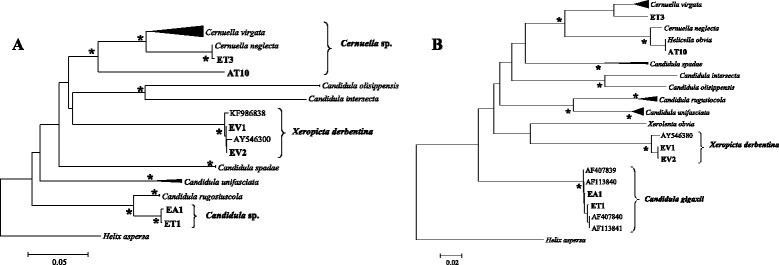
**Phylogenetic trees based on COI (A) and 16S (B) sequences using the Maximum Likelihood method and the Tamura Nei model (T92 + Γ).** The trees have been rooted using *Helix aspersa* (HQ203052 and HM627384). The asterisk indicates bootstrap values of >90 for NJ, ML and ME.

An initial study of the combined dataset of 18S, ITS-1, COI and 16S sequences show the membership of all our positive snails to the Hygromiidae family (Figure 3) and the separation in three different clades corresponding to the different morphotypes. A second study, by analysing the specific 16S and COI allows us to identify some of the species involved in the different groups (Figure 4). Groupe A is only composed of the species *Xeropicta derbentina* Brusina, 1870 and group C of *Candidula gigaxii* (Pfeiffer, 1850). For group B molecular analyzes was not conclusive at specific level. Both haplotypes differ between groups. Estimation of the pairwise distances with other genera (70-75% of homology) is in favor of their membership to the genus *Cernuella*, thus confirming the results obtained during the morphological identification. Among the *Cernuella* genus, the pairwise distances with other species do not separate specifically between the two haplotypes, with percentages of homology very close to several species such as *Cernuella virgata*, *C. neglecta* and *C. Cisalpina*, without consistency between the analyzed domain (16S or COI).

## Discussion

Lungworm infections are frequently found in hares in France [41], but few studies have been conducted to look for intermediate hosts of these parasites in natural conditions. This approach is, however, essential for understanding the development of the parasite life cycle, and thus to evaluate the risk of dispersion and contamination of the parasite in populations of definitive hosts. Our study aims to find these hosts *in natura* for different species of protostrongylids previously isolated from lungs of hares in France, in order to identify the host-parasite relationship and more precisely the gastropod-nematode relationship, by using techniques previously tested for Trematodes [64-66].

Our research of potential intermediate hosts (Gastropods) was therefore carried out on the sites where positive hares had been located. It is important to note that the collection method used here is not suitable for small snail species approx a millimeter in size, living in ground litter and moving very little and very slowly, and already identified as an intermediate host of Protostrongylidae, e. g: *Euconulus fulvus* or *Vertigo alpestris* [28]. Hence we cannot exclude the possibility that we missed some small snail species infested by Protostrongylids, meaning that the number of intermediate host species could be higher than five*.* Among the 3622 gastropods isolated, no slug was found positive after experimental infection, even if some specimens were previously reported as intermediate hosts. For terrestrial snails, eight different families were isolated, but only snails belonging to the Hygromiidae were found positive in natural conditions for Protostrongylids larvae. Other families of snails among the Pulmonata have been referenced in the literature, but always experimentally [1,27,28]. The method used to search for larvae in molluscs is different from that usually performed. If using pepsin-digestion is commonly done [67-70], especially for large molluscs, we prefer the pressing method of snails between two glass plates for small specimens. We do not compare these two methods here. The proportion of infested snails is low (0.54%) but similar to other previous studies [13,71,72]. The number of larvae per snail varied from 1 to 52 with an average of 7 larvae per snail. The mortality rate is higher among the snails which harbour a considerable number of *Neostrongylus linearis* than the non-infected or moderately infected molluscs [73]. We can suppose that heavily infested individuals have not been collected during this study. Molecular analyzes of the larvae confirm the presence of two species of *Protostrongylus*, *P. oryctolagi* and *P. pulmonalis*, the same species as those previously found on hares in France.

The analysis of the positive snails based on the morphological criteria of the shell highlighted 4 groups, limited at the family or the genus level. Identification at the specific level is extremely difficult because of the important intra and inter-specific polymorphism. To be free from the constraints related to morphological identification, we used molecular tools, as previously described, to identify species incriminated as intermediate hosts [54,55,60].

Larvae of *P. pulmonalis* was isolated from one individual belonging to the *Xeropicta derbentina* species. Larvae of *P. oryctolagi* were observed in four different species of snails: *Candidula gigaxii* (Pfeiffer, 1850)*, Xeropicta derbentina* Brusina, 1870 and two *Cernuella* sp. If molecular results confirm the monophyly of the genus *Cernuella*, the haplotype *Helicella obvia* sensu Di Napoli et al. [54] most probably belong to the species *Cernuella neglecta* [74], we will not conclude at the specific level for both haplotypes isolated during our study. In fact, due to the high intra-specific morphological variability for this genus, sometimes including the internal features, identification is often very difficult. Under these conditions and based on the ambiguous molecular results, we consider these two haplotypes as *Cernuella* sp. pending a more comprehensive study.

This is the first report of all these snail species as natural intermediate host of *Protostrongylus* of Lagomorphs, except *Xeropicta derbentina* already infested experimentally by *Protostrongylus tauricus* [1].

Protostrongylidae are not highly specific in their use of intermediate hosts since many species of snail have been shown to be involved in its development [32,75]. The specificity between the nematode parasite and its intermediate host seem less strict compared to what can be observed especially for trematodes. However only some intermediate host species are probably important in natural transmission to the definitive host. *Candidula gigaxii, Cernuella* spp. and *Xeropicta derbentina* mainly colonize open fields with dry environment and calcareous soil typical of the landscape in the Mediterranean area [63,76], with a large part of French territory and some European countries. Introduced from East of Europe in 1940, *X. derbentina* is limited in most open environments in Provence, mainly pasture fields and vineyards, where populations of snails are often very dense [77-79].

In our study, all larvae of *Protostrongylus* were isolated from snails collected in wine areas. All four species of intermediate hosts were also observed in grassland areas, but their abundance seems lower. It is possible that the wine crops are better adapted to the environment allowing snails to release larvae or offer optimal conditions for larvae survival. Third-stage larvae of *Muellerius capillaris* remain infective for at least six months at −12°C [80], but are supposed to be very sensitive to dry conditions [81] and the wine areas, drier than grassland, do not seem best suited for larval survival. However, third-stage larvae, which are infective for the definitive host by ingestion, do not usually leave the foot when the snail is alive but their output is activated when it dies [75]. We can suggest that chemical treatments used in wine crops could modulate levels of parasitic infestation in snail populations. Indeed, Hock and Poulin [82] observed that pollutants can lead to increase cercariae excretion from aquatic snails.

An experimental study should be initiated to verify whether larvae excretion is facilitated by use of treatments, causing intense drooling and death by desiccation of snails.

## Conclusion

Using a combined morphological and molecular approach for parasites and snails, this study displays the first report of natural intermediate hosts of *P. oryctolagi* and *P. pulmonalis*, two parasitic nematodes responsible for pulmonary protostrongyliasis in France, disease frequently encountered in hare populations in Europe*.* Understanding the life cycle of this kind of parasites is essential for risk factor identification. Among the two different habitats sampled, the wine crops seem favourable to the life cycle of hare *Protostrongylus*. In the future, it would be interesting to implement investigation in other environments in order to identify all habitats representing potential risks of disease for hare populations.
